# Electroacupuncture Relieves HuR/KLF9-Mediated Inflammation to Enhance Neurological Repair after Spinal Cord Injury

**DOI:** 10.1523/ENEURO.0190-23.2023

**Published:** 2023-11-20

**Authors:** Junfeng Zhang, Jingjie Xu, Shisheng Li, Wei Chen, Yaochi Wu

**Affiliations:** Department of Acupuncture, Tuina and Traumatology, The Sixth People's Hospital Affiliated to Shanghai Jiaotong University, Shanghai 200233, People’s Republic of China

**Keywords:** electroacupuncture, HuR, KLF9, neurological repair, SCI

## Abstract

Electroacupuncture (EA) is widely applied in clinical therapy for spinal cord injury (SCI). However, the associated molecular mechanism has yet to be elucidated. The current study aimed to investigate the underlying mechanism of EA in neurologic repair after SCI. First, we investigated the role of EA in the neurologic repair of the SCI rat model. The expression levels of human antigen R (HuR) and Krüppel-like factor 9 (KLF9) in spinal cord tissues were quantified after treatment. Second, we conducted bioinformatics analysis, RNA pull-down assays, RNA immunoprecipitation, and luciferase reporter gene assay to verify the binding of HuR and KLF9 mRNA for mRNA stability. Last, HuR inhibitor CMLD-2 was used to verify the enhanced effect of EA on neurologic repair after SCI via the HuR/KLF9 axis. Our data provided convincing evidence that EA facilitated the recovery of neuronal function in SCI rats by reducing apoptosis and inflammation of neurons. We found that EA significantly diminished the SCI-mediated upregulation of HuR, and HuR could bind to the 3′ untranslated region of KLF9 mRNA to protect its decay. In addition, a series of *in vivo* experiments confirmed that CMLD-2 administration increased EA-mediated pain thresholds and motor function in SCI rats. Collectively, the present study showed that EA improved pain thresholds and motor function in SCI rats via impairment of HuR-mediated KLF9 mRNA stabilization, thus providing a better understanding of the regulatory mechanisms regarding EA-mediated neurologic repair after SCI.

## Significance Statement

Although electroacupuncture (EA) has been used to treat spinal cord injury (SCI) in recent years, its regulatory mechanism has not yet been clarified. To elucidate the molecular mechanism of EA in reducing SCI, we conducted a series of *in vivo* and *in vitro* experiments. EA contributes to neurologic repair of SCI rats through diminishing the SCI-mediated upregulation of human antigen R (HuR) and Krüppel-like factor 9 (KLF9). Additionally, HuR, the downstream target of EA, physically interacted with the 3′ untranslated region of KLF9 mRNA to protect its decay. These findings provide a connection between EA and neuron repair. Furthermore, this study also provides a theoretical basis for EA in the clinical treatment of SCI.

## Introduction

Spinal cord injury (SCI) is a severe damage to the spinal cord that results in the loss of sensory and motor abilities depending on the site of the injury (Hawasli et al., 2018). Etiologically, accidental traumatic events >90% of SCI cases (Feigin et al., 2019). The global incidence of SCI is increasing, with ∼10.4–83 cases per million reported each year (Karsy and Hawryluk, 2019). Within several minutes, acute traumatic injuries to the neural tissues and vessels can result in a series of other primary injuries, including ischemia, hypoxia, edema, and glutamate excitotoxicity (Zhang et al., 2021). Additionally, primary injuries can induce secondary injuries characterized mainly by inflammation, with activated microglia, neutrophils, peripherally derived macrophages, lymphocytes, and pericytes (Hellenbrand et al., 2021). Consequently, cellularly distinct compartments composed of a fibrotic core, a border of astrocytes, and adjacent reactive neural tissue can be formed during the secondary injuries to strongly inhibit the regeneration of axons (Anderson et al., 2022). Inflammatory reaction after SCI is thus the most important pathologic process to address during secondary injuries. Controlling excessive inflammation has been shown to improve the prognosis of SCI both clinically and in animals, as evidenced by accumulating reports (Gillespie and Ruitenberg, 2022; Zhang et al., 2022; Zhou et al., 2022b).

Acupuncture is an ancient Chinese medical technique that has been used to treat various neurologic diseases, such as Parkinson’s disease (Zhao et al., 2021), Alzheimer’s disease (Yin et al., 2021), and epilepsy (Deng et al., 2018). Electroacupuncture (EA) is a specific type of acupuncture that involves the use of a small electrical current passing with pairs of needles (Zhuang et al., 2013). EA was implied to provide enhanced benefits to recovery of neurologic function and improvement of motor function compared with manual acupuncture (MA), as EA showed better outcomes in SCI rats treated at Shuigou (DU26) and Fengfu (DU16; Jiang et al., 2014). Jiang et al. (2014) have reported that EA stimulation leads to neuroprotection and neuronal function recovery in SCI rats, mainly because of the anti-inflammatory function of acupuncture. The molecular mechanism associated with the regulation of inflammatory response by EA have been revealed in various diseases, such as the mucin 5AC/EGFR/p38MAPK signaling pathway in chronic obstructive pulmonary disease (Lin et al., 2021), and the TGF-β1/Smad3/p38/ERK1/2 pathway in skeletal muscle inflammation (Han et al., 2021). However, the anti-inflammatory signaling pathway associated with EA in neurologic repair after SCI has yet to be delineated.

Human antigen R (HuR) is an RNA-binding protein that enhances the stability and translation efficiency of target mRNA by binding to its 3′ untranslated region (UTR) region. It has been implied to play a critical role in nervous system diseases. Higher levels of phosphorylation and protein expression of HuR have been observed in the motor cortex and spinal cord of amyotrophic lateral sclerosis patients (Milani et al., 2013). The main behavioral symptoms could be completely reversed by intrathecal administration of anti-HuR antisense oligonucleotide in an autoimmune encephalomyelitis model (Sanna et al., 2017). Previous research has demonstrated that HuR overexpression in astrocytes accentuates SCI (Kwan et al., 2017b). Despite numerous *in vitro* studies, specific HuR-associated mechanisms in neurologic repair after SCI remain to be unraveled through *in vivo* experiments.

Krüppel-like factor 9 (KLF9), a transcription factor of the KLF family, is responsible for maintaining the necessary synaptic changes that are required for neuronal sensitization and pain in the spinal cord network (Mamet et al., 2017). Altered expression of KLF9 has been observed both in the α-synuclein (α-syn) mouse model and α-syn exposed cellular model of Parkinson’s disease (Lin et al., 2022). Furthermore, KLF9 is also involved in thyroid hormone-activated pathways for neuronal protection and the promotion of neuronal recovery after injury (Li et al., 2017a). Although the important roles of *KLF9* in neurologic diseases have been revealed, its detailed function and the underlying mechanism in SCI remain unclear.

There is a lack of data regarding the effect of EA on the HuR/KLF9 pathway in SCI and its associated mechanisms. In the present study, we investigated the effects of EA on the neurologic recovery of SCI rats and the possible HuR-associated mechanisms, which might provide novel perspectives for SCI research.

## Materials and Methods

### Spinal cord neurons cell culture

Ventral spinal cord 4.1 (VSC4.1) motoneuron cell line (catalog #MZ-0784, Ningbo Mingzhou Biotechnology Co, Ltd.) was grown in 75 cm^2^ flasks precoated with 0.01% poly-l-ornithine (PLO; catalog #P3655, Merck KGaA) and cultured in high-glucose DMEM (catalog #11965092, Thermo Fisher Scientific) supplemented with 15 mm HEPES (catalog #15630130, Thermo Fisher Scientific), pyridoxine NaHCO_3_ (catalog #S6014, Sigma-Aldrich), 2% Sato’s components (self-made), 1% penicillin and streptomycin (catalog #15140148, Thermo Fisher Scientific) as well as 10% fetal bovine serum (catalog #10100, Thermo Fisher Scientific). The cells in our study were incubated at 37°C in a humidified 5% CO_2_ incubator.

### Establishment of SCI model

Male adult Sprague Dawley rats (age range, 7–8 weeks; weight range, 250–300 g) were purchased from the Experimental Animal Center of Shanghai Jiaotong University [permit SYXK (Shanghai) 2018-0028], and all animal studies were approved by the Animal Experimental Committee of The Sixth People’s Hospital [permit SCXK (Shanghai) 2018-0006]. The rats were randomly divided into the sham group, SCI group, EA group, and EA+CMLD-2 group (*n* = 10/group); were weighed; and were intraperitoneally injected with 4% chloral hydrate (10 ml/kg). Then a laminectomy was performed at the T9–T10 level to expose the cord beneath protecting the dura mater. The New York University Impactor System (NYU Impactor) was used to perform the spinal card contusion on the exposed dorsal surface (10 g × 25 mm; Yune et al., 2007). The performance of strong lower limb contraction and tail-spasm swing was used as indicators for the successful establishment of rat SCI model. A T10 laminectomy without weight-drop contusion injury was performed on rats in the sham group, and the incision was sutured layer by layer. For CMLD-2 treatment, CMLD-2 (30 μm; catalog #538339, Millipore) was dissolved in DMSO (catalog #D1435, Sigma-Aldrich) at a final concentration of 10 mm and further diluted to 2 mm in the mother liquor solution with glycerol. After inducing the injury in the SCI model for 24 h, the rats were injected with CMLD-2 (10 mg/kg) intrathecally once every 3 d for 1 week to ensure HuR inhibition. The penicillin sodium (20,000 U; catalog #P0389, Sigma-Aldrich) was administered through intramuscular injection in the hindlimb once daily for the first 3 d after surgery to avoid infection. Bladders of all rats that underwent SCI were emptied manually three times a day until normal micturition reflex was developed. After all tests were completed, the Sprague Dawley rats were intraperitoneally anesthetized with 3% pentobarbital sodium (30 mg/kg; catalog #11715, Sigma-Aldrich), then spinal cord at T10 (10 mm long segment) was uncovered through a dorsal laminectomy. After stripping blood vessels and spinal meninges, the spinal cord tissue was obtained. All surgical interventions and postoperative animal care were performed according to the guidelines and policies of the Animal Experimental Committee of The Sixth People's. Hospital [permit SCXK (Shanghai) 2018–0006].

### EA treatment

EA was performed at the following six acupoints: Dazhui [Governing Vessel 14 (GV14); between the seventh cervical vertebra and the first thoracic vertebra, in the middle of the back]; Shengmen (GV4; on the midline of the back, the depression under the spinous process of the second lumbar spine); Shuigou (GV26; the center of 1 mm below the nose tip); Yanglingquan [Gall Bladder 34 (GB34); on the outside of the lower leg, in the anterior depression of the fibular head; ∼3 mm from the upper and outer side of Zusanli]; Baihui (GV 20; center of parietal bone); and Jizhong (GV6; between the 11th thoracic spine and the 12th thoracic spine) after 24 h of inducing SCI by inserting disposable sterile acupuncture needles (13 × 0.3 mm; catalog #6901452122118, Hwato) at a depth of 20 mm (Choi et al., 2010; Cheng et al., 2020). Then, an electric stimulator (Han’s acupoint nerve stimulator; model HANS-200, Han Institute) was used to stimulate the acupoints for 30 min (current intensity, 2 mA; wave, 50 Hz; Cheng et al., 2020). EA treatment was given once a day for 1 week to the EA group, whereas the sham and SCI groups without any EA treatment were simulated with a toothpick at each acupoint as previously described (Choi et al., 2010).

### Determination of mechanical withdrawal threshold and thermal withdrawal latency

To evaluate the effect of EA or/and CMLD-2 treatments on pain threshold of SCI rats, mechanical withdrawal threshold (MWT) and thermal withdrawal latency (TWL) were measured to assess the thermal nociception and hyperalgesia of every rat on day 8 post-SCI. To calculate MWT, von Frey filaments (catalog #NC12775, Stoelting; 2, 4, 6, 8, and 15 × *g* for ≤4 s) were used to stimulate the metatarsal surface of the rats five times (Li et al., 2017b). A positive foot withdrawal response was scored if the rat raised the stimulated claw. A threshold of 50% positive response was determined using the up-down method (Bao et al., 2015) for MWT and TWL measurements. To calculate TWL, the outer sole of the rat foot was exposed to the hot plate (50°C), and latency from the heat onset to a brisk withdrawal was measured three times at 2 min intervals in the center of the paw.

### Basso, Beattie, and Bresnahan scores

To evaluate the effect of EA or/and CMLD-2 treatments on the neurologic function of SCI rats, Basso, Beattie, and Bresnahan (BBB) scores were measured after EA or/and CMLD-2 treatments according to the previously described method (Choi et al., 2010). Briefly, rats were placed on a 1 m round plane and observed for 3 min by two trained researchers simultaneously. A 22-point scale (scores, 0–21) was used that systematically and logically follows the recovery of hindlimb function from a score of 0, representing no observed hindlimb movements, to a score of 21, representing normal gait.

### Footprint analysis

To confirm the effect of EA or/and CMLD-2 treatments on the motor function of SCI rats, footprint analysis was performed by using a 5-point scale (Metz et al., 2000). Briefly, the hindlimbs of the rats were dipped in ink and then walked across a narrow (60 × 7.5 cm) dark channel. The other side of the dark channel was kept transparent for the guide rats to move in the same direction, and the bottom of the channel was covered with test paper to record the footprints. The test was performed after EA or/and CMLD-2 treatments. Scores were evaluated by taking six consecutive footprints per group, with a score of 0, indicating continuous touchdown of foot-back and dragging of the posterior limb, and a score of 5, indicating no rear footprints of plantar.

### Inclined plane test

To confirm the effect of EA or/and CMLD-2 treatments on the motor function of SCI rats, an inclined plane test was performed in rats after EA or/and CMLD-2 treatments to evaluate the residual strength of the forelimbs and hindlimbs after injury and the ability to maintain body balance. In brief, a rat was placed on a movable inclined plane and was tilted gradually from 1° to 52°. The rat was required to maintain its position for 5 s without falling at each angle. The maximum angle was recorded, and a single average score was obtained for each animal.

### Swimming test

To confirm the effect of EA or/and CMLD-2 treatments on the motor function of SCI rats, the swimming test (Hara et al., 2000) was conducted in a cube glass container that was 150 × 15 × 40 cm in size. Warm water was poured into the container, and an inclined rectangular mirror was placed at the bottom to record foot movement during swimming with a camera. A T-shaped platform was installed in the swimming direction with a swimming distance of 60 cm. The test video of each rat was recorded by a high-definition camera, and the swimming speed, forelimb flutter, tail swing, and hindlimb position were analyzed. The experiment was conducted after EA or/and CMLD-2 treatments. All tested rats were trained twice a day for 1 week before operation to cultivate their directionality and eliminate stress reactions.

### TUNEL staining and 5-ethynyl-2'-deoxyuridine staining

To determine the situation of cell apoptosis, the tissue sections were fixed in 4% paraformaldehyde solution and stained with TUNEL fluorescence dye to assess cell death using an In Situ Cell Death Detection Kit (catalog #S7100, Roche Diagnostics) according to the manufacturer guidelines. At least three photographs from random fields of each stained section were captured and analyzed with Image-Pro Plus 6.0 (Media Cybernetics).

To determine the situation of neuronal proliferation, 5-ethynyl-2'-deoxyuridine (EDU) staining was performed using Click-iT EdU Imaging Kit (Thermo Fisher Scientific) according to the manufacturer protocol. The tissue sections were fixed with 4% paraformaldehyde for 15 min. After washing twice with bovine serum albumin (BSA; 3%), the sections were permeabilized with Triton X-100 (0.5%) for 20 min. Then sections were again washed twice and incubated with a Click-iT reaction cocktail and reaction buffer additive for 0.5 h while protected from light. The sections were washed once more with 3% BSA in PBS. Subsequently, sections were incubated with Hoechst 33342 stain (5 μg/ml) for 0.5 h. The percentage of EDU-positive/Hoechst-positive cells was calculated using Image-Pro Plus version 6.0 (Media Cybernetics).

### Hematoxylin and eosin and immunohistochemical staining

To determine the pathologic damage of spinal cord tissue, the spinal cord tissue samples were collected and fixed with 4% paraformaldehyde for 24 h. After dehydrating with gradient ethanol, the sections (thickness, 8 µm) were stained using hematoxylin and eosin (H&E) or related antibodies according to instructions, and the histopathology was assessed using a light microscope (model TE300, Nikon). The following antibodies were used: anti-HUR (1:300; catalog #39–0600, Thermo Fisher Scientific); anti-KLF9 (1:150; catalog #OTI8A11, Novus Biologicals); and goat anti-mouse IgG (1:200; catalog #ab150113, abcam). The comprehensive score (range, 0–12) is obtained by multiplying the scored values of the staining intensity (range, 0–3), and the positivity rate (range, 0–4) in immunohistochemical slices. Specifically, the scoring of staining intensity is based on the staining characteristics of the target cells: no staining is scored as 0; light yellow staining is scored as 1; light brown staining is scored as 2; and brownish-brown staining is scored as 3. The scoring based on the cell positivity rate is as follows: 0–5% is scored as 0; 6–25% is scored as 1; 26–50% is scored as 2; 51–75% is scored as 3; and >75% is scored as 4.

### ELISA

To analyze the levels of inflammatory factors, the levels of cytokines TNF-α, IL-1β, and IL-6 in spinal cord tissues were quantified with ELISA kits (Beyotime Institute of Biotechnology, Haimen, People’s Republic of China) according to the manufacturer instructions. The kits used were rat TNF-α ELISA Kit (catalog #PT516, Beyotime Institute of Biotechnology), rat IL-1β ELISA Kit (catalog #PI303, Beyotime Institute of Biotechnology), and rat IL-6 ELISA Kit (catalog #PI328, Beyotime Institute of Biotechnology).

### Analysis of mRNA binding to HuR

To determine the downstream targets of HuR, we conducted bioinformatics analysis through four databases, included RNAInter (http://www.rnainter.org/), ENCORI (https://rnasysu.com/encori/), RBPTD (http://www.rbptd.com/#/), and POSTAR3 (http://111.198.139.65/). Using the Venn diagram (https://bioinfogp.cnb.csic.es/tools/venny/index.html), we obtained the intersection of four databases for predicting mRNA. Afterward, the binding sites on the predicted mRNA targets or HuR protein were assessed using PRIdictor (http://rnainter.org/PRIdictor/) from RNAInter.

### RNA extraction and quantitative real-time PCR

To determine the abundance of HuR and KLF9 mRNA, total RNA was extracted from the spinal cord tissue of rats using the TRIzol reagent (catalog #15596026CN, Thermo Fisher Scientific). cDNA was synthesized by reverse transcription using a First Strand cDNA Synthesis Kit (catalog #6210A, TaKaRa) according to the manufacturer instructions. Relative levels of mRNA expression were calculated by the 2^–ΔΔCt^ method (Livak and Schmittgen, 2001), quantified using the Power SYBR Green Master Mix (catalog #A25780, Applied Biosystems), and normalized to the expression levels of GAPDH. Primers used are listed as follows: HuR, forward: 5'-GTT AGA CAG ATG GGG ACT GTG T-3’; HuR, reverse: 5'-AAC ACC ATA GGG CTG GGA CT-3’; KLF9, forward: 5'-CCT TGA GGG TAG AAG CCG AC-3’; KLF9, reverse: 5'-AAA AGG GGG CAT TTC CAG GT-3’; GAPDH, forward: 5'-TGT GAA CGG ATT TGG CCG TA-3’; and GAPDH, reverse: 5'-GAT GGT GAT GGG TTT CCC GT-3′.

### Western blotting

To determine the protein levels of Caspase-3, HuR, and KLF9, Western blot was performed. The tissue was homogenized in 0.2 ml of homogenization solution and centrifuged for 10 min. Equivalent amounts of protein from the supernatant were separated on SDS-PAGE gels (catalog #P0012A, Beyotime Institute of Biotechnology) and transferred to PVDF membranes (150 mA, 1.5 h; catalog #GVWP02500, Millipore), and blocking was done with 5% skim milk for 1 h. The membranes were incubated overnight with different primary antibodies at 4°C, including anti-Caspase-3 (1:1000; catalog #9664, Cell Signaling Technology), anti-HuR (1:000; catalog #39-0600, Thermo Fisher Scientific), anti-KLF9 (1:1000; catalog #gtx129316, GeneTex), and anti-GAPDH (1:1000; catalog #92310, #8986, Cell Signaling Technology). The membranes were then washed and incubated for 2 h in peroxidase-conjugated rabbit anti-goat IgG secondary antibody (1:1000; catalog #A5420, Sigma-Aldrich) at room temperature. The membranes were washed three times for 10 min and incubated with enhanced chemiluminescence detection reagents. The detected protein bands were analyzed using AlphaEaseFC software (Alpha Innotech).

### RNA immunoprecipitation

To confirm the interaction between HuR and KLF9 mRNA, cells were collected and incubated with 1 ml of precooled RNA immunoprecipitation (RIP) Lysis Buffer (catalog #P0013B, Beyotime Institute of Biotechnology; 4°C, 5 min). The cell suspension was centrifuged at 4°C for 10 min, and the supernatant was collected, added to 20 µl of protein A/G magnetic beads (catalog #26162, Thermo Fisher Scientific), and discarded after stabilizing for 10 s. After that, cells were coimmunoprecipitated with anti-HuR mouse antibody (1:500; catalog #sc-5261, Santa Cruz Biotechnology) or normal anti-IgG mouse antibody (1 µg/100 µg of total protein; catalog #sc-374321, Santa Cruz Biotechnology), and incubated at room temperature for 30 min. After centrifugation and rinse with RIP wash buffer, the TRIzol reagent was appended to immunoprecipitated RNA and the interaction between protein and RNA was determined through quantitative real-time PCR (qRT-PCR).

### RNA pull-down assay

To confirm the binding of HuR and KLF9 mRNA, KLF9 was transcribed *in vitro* with the T7 RNA polymerase (catalog #AM2085, Ambio Life), purified using the RNeasy Plus Mini Kit (catalog #74136, Qiagen), and added with DNase I (catalog #18047019, Thermo Fisher Scientific). A fresh cell lysis buffer was prepared using the Pierce Magnetic RNA-Protein Pull-Down Kit (catalog #20164, Thermo Fisher Scientific). Next, the extracted cellular proteins were cultured with biotinylated RNA probes targeting KLF9 and added to magnetic beads for binding reaction. The eluted HuR protein was further validated by Western blot analysis.

### Dual-luciferase assay

To determine the binding of HuR and the 3′ UTR region of KLF9 mRNA, dual-luciferase reporter plasmid (GeneCopoeia) containing wild-type (WT) 3'-UTR sequence of KLF9 mRNA or a mutant sequence were transfected into 293T cells along with shRNA targeting HuR (shHuR) or the negative control (NC). After 48 h, firefly and Renilla luciferase activities were measured using a dual-luciferase assay kit (catalog #E1960, Promega); Renilla luciferase activity signal was used for the normalization.

### Statistical analysis

Statistical analysis of the data were performed using SPSS 23.0 software (SPSS), and data were presented as the mean ± SD. Student’s *t* test was used to analyze the differences between two groups. One-way ANOVA followed by the Tukey’s test was used for multisample analysis; *p* < 0.05 was considered statistically significant.

### Data availability

All data in our study are available upon request.

## Results

### EA treatment enhanced neurologic repair after SCI

**Figure 1.**
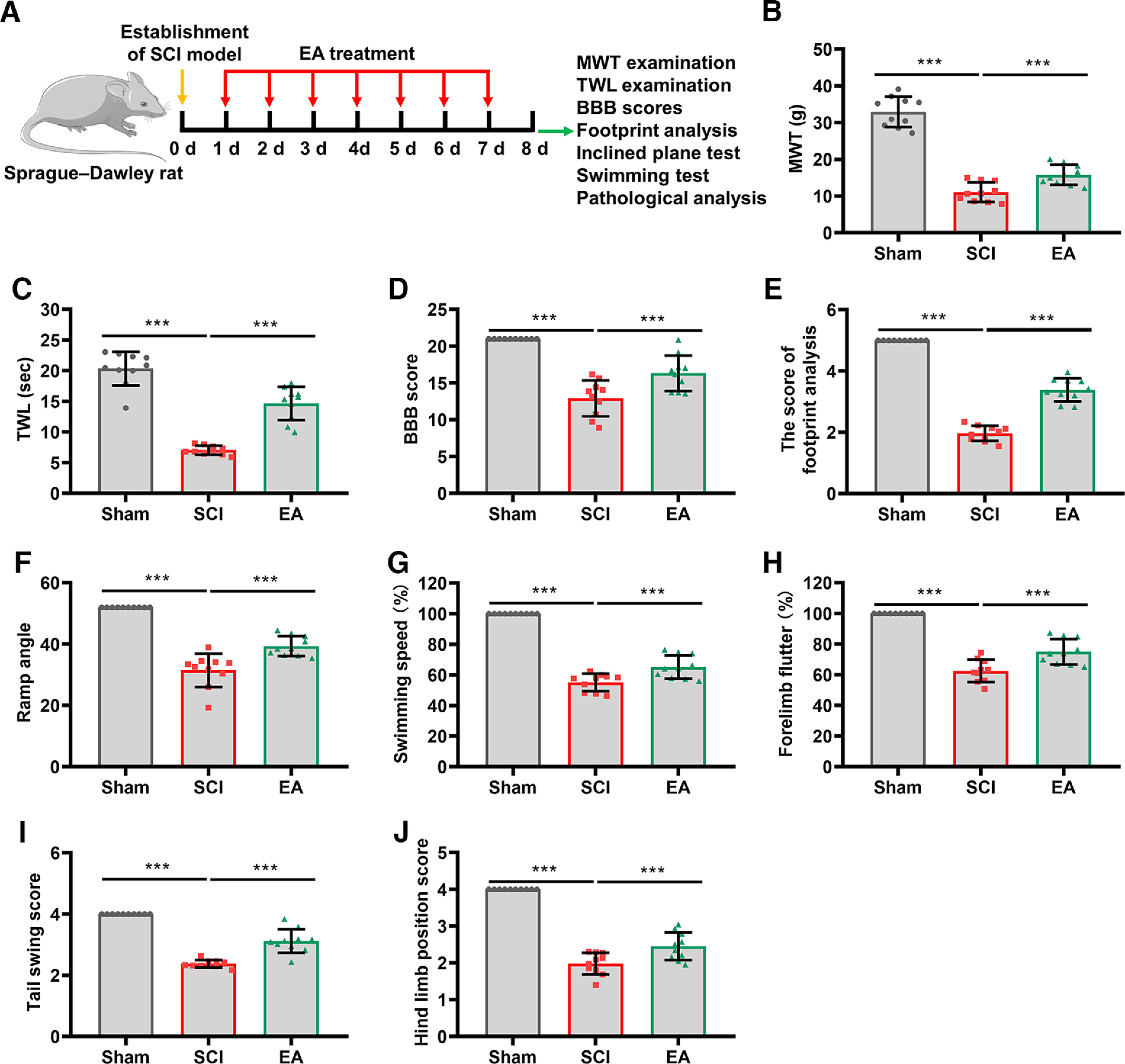
EA treatment contributed to the neurologic function recovery of SCI rats. ***A***, Flowchart of the EA treatment given to SCI rats. Neurogenic behaviors were tested and spinal cord samples were harvested at day 8. ***B***, ***C***, The effects of EA treatment on SCI-induced hyperalgesia were evaluated by MWT and TWL (*N* = 10). ***D***, The neurologic function of SCI rats treated with or without EA were assessed BBB scores (*N* = 10). ***E***, The footprint test was performed to assess the movement stability and balance ability of the rats after sham surgery, SCI operation, or EA treatment (*N* = 10). ***F***, The SCI rats with or without EA treatment were analyzed using the inclined plane test, and the angle was recorded (*N* = 10). ***G–J***, Swimming performance of the rats in the Sham, SCI, and EA groups was evaluated by swimming rate (***G***), forelimb flutter (***H***), tail swing score (***I***), and hindlimb position **(*J*)** to assess motor function recovery (*N* = 10). The *p* values were calculated with Tukey’s test for ***B–J***. ***p < 0.001.

GV26 and GB34 have been characterized as the most potent anti-inflammatory acupoints in the SCI treatment (Choi et al., 2010). EA stimulation with a low frequency at GV14 and GV4 was implied to relieve SCI via inhibiting oxidative stress, inflammation, and apoptosis in rats (Cheng et al., 2020). And GV20 and GV6 were recommended in a clinical study, as the acupoints could improve the therapeutic effects compared with those whose acupoints were chosen along the injured nerve trunk (He et al., 2015). In this study, we explored the impact of EA treatment with these acupoints in the neurologic repair of rats with SCI (Fig. 1*A*). First, we recorded MWT and TWL values to assess the effectiveness of EA treatment on SCI-induced hyperalgesia (Fig. 1*B*,*C*). The results indicated that the MWT and TWL values of the SCI group were significantly lower than those for the sham group. However, EA treatment in the SCI rats notably increased the MWT and TWL values (*p *<* *0.001, *n* = 10), implying that EA increased the pain thresholds in the SCI rats. The effects of EA on the neurologic function of rats were quantified by the BBB locomotor scale. As presented in Figure 1*D*, lower BBB scores were observed in the SCI group compared with the sham group, and EA treatment significantly improved BBB scores (*p *<* *0.001, *n* = 10). Further, footprint analysis was performed to study the effects of EA on the movement stability and balance ability of the rats. We found that the footprint score in the SCI group was significantly less than in the sham group, whereas EA treatment increased the footprint score in SCI rats (*p *<* *0.001, *n* = 10), indicating a significant recovery of motor function by EA (Fig. 1*E*). An inclined plane test was performed to further evaluate the locomotor recovery of rats in each group. Rats in the SCI group maintained 30° on the inclined plane for 5 s, but EA treatment made SCI rats hold longer on steeper inclined planes (*p *<* *0.001, *n* = 10; Fig. 1*F*). We further determined the swimming performance of each rat by evaluating the following four indicators: swimming rate, forelimb flutter, tail swing score, and hindlimb position. As shown in Figure 1*G*, EA treatment partially rescued decreased swimming rate in the SCI rats (*p *< 0.001, *n* = 10). In addition, the decreased forelimb flutter frequency and tail swing scores in response to SCI-induced damage in rats were partially rescued by EA treatment (*p *<* *0.001, *n* = 10; Fig. 1*H*,*I*). Furthermore, we also found that the hindlimbs of rats who received EA stimulation were significantly closer to the central axis of the body during swimming, whereas the hindlimbs of rats in the SCI group were relatively far away from the body (*p *<* *0.001, *n* = 10; Fig. 1*J*). These findings confirm that EA treatment at GV26, GB34, GV14, GV4, GV20, and GV6 acupoints enhances neurologic repair in rats after SCI.

### EA treatment relieved inflammation after SCI

**Figure 2.**
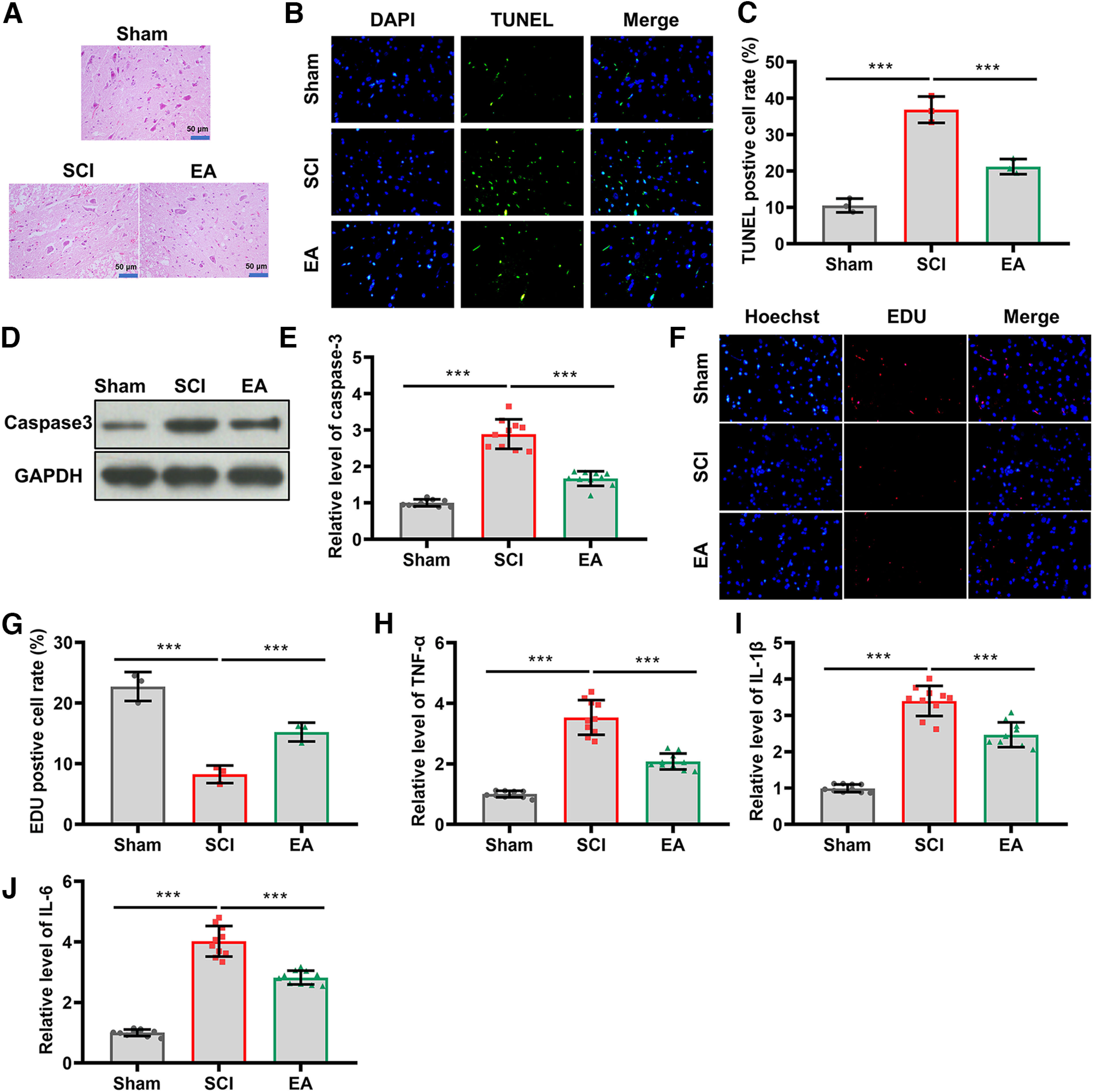
EA treatment relieved inflammation in SCI rats. ***A***, H&E staining was used to assess the damage to spinal cord tissues of the rats in the Sham, SCI, and EA groups (*N* = 3). Scale bar, 50 µm. ***B***, TUNEL staining was used to detect cell apoptosis in spinal tissue sections from each group (*N* = 3, ×100). ***C***, Statistical comparison of the levels of TUNEL-positive cells (*N* = 3). ***D***, EDU staining was used to detect cell proliferation in spinal tissue sections from each group (*N* = 3, ×100). ***E***, Statistical comparison of the levels of EDU-positive cells (*N* = 3). ***F***, Western blotting was performed to measure Caspase-3 protein expression in the spinal cord tissues of the rats after sham surgery, SCI operation, or EA treatment (*N* = 10). ***G***, Statistical comparison of Caspase-3 protein levels in each group (*N* = 10). ***H*–*J***, ELISA was performed to measure the levels of TNF-α, IL-1β, and IL-6 in the Sham, SCI, and EA groups (*N* = 10). The *p* values were calculated with Tukey’s test for ***D–G***. ***p < 0.001.

The acupoints were selected for their ability to inhibit inflammation and oxidative stress, and to enhance therapeutic effects (Choi et al., 2010; He et al., 2015; Cheng et al., 2020). However, it remains to be investigated whether this combination of EA will lead to better outcomes. To determine the neuroprotective role of EA, spinal cord tissues were stained with H&E. The results indicated that central gray matter and dorsal white matter of SCI rats were seriously damaged compared with the sham group, while EA treatment attenuated these damages (Fig. 2*A*). Next, we examined the reduction in cell apoptosis by EA treatment through TUNEL-positive neuron counting. As shown in Figure 2, *B* and *C*, EA treatment prevented apoptosis of neurons induced by SCI. Further, Western blot analysis showed that SCI resulted in a significant upregulation of Caspase-3 protein in spinal cord tissues compared with the sham group (*p *<* *0.001), but EA treatment reversed this effect (*p *<* *0.001, *n* = 10; Fig. 2*D*,*E*). Moreover, we detected the proliferation of neurons and found that EA treatment contributed the proliferation of neurons induced by SCI (*p *<* *0.001, *n* = 10; Fig. 2*F*,*G*). Subsequently, we determined the expression of inflammatory molecules, including TNF-α, IL-6, and IL-1β in spinal cord tissues through ELISA to study the potential effects of EA on SCI-induced inflammation. Compared with the sham group, SCI increased TNF-α levels in the damaged spinal cord, whereas EA reversed this increase (*p *<* *0.001, *n* = 10; Fig. 2*H*). In addition, EA stimulation restored the elevated levels of IL-6 induced by SCI (*p *<* *0.001, *n* = 10; Fig. 2*I*). Upregulation of IL-1β induced by SCI was also reversed by EA stimulation (*p *<* *0.001, *n* = 10; Fig. 2*J*). Overall, these results indicated that EA treatment at GV26, GB34, GV14, GV4, GV20, and GV6 acupoints relieved inflammation after SCI.

### EA treatment regulated KLF9-mediated HuR expression after SCI

**Figure 3.**
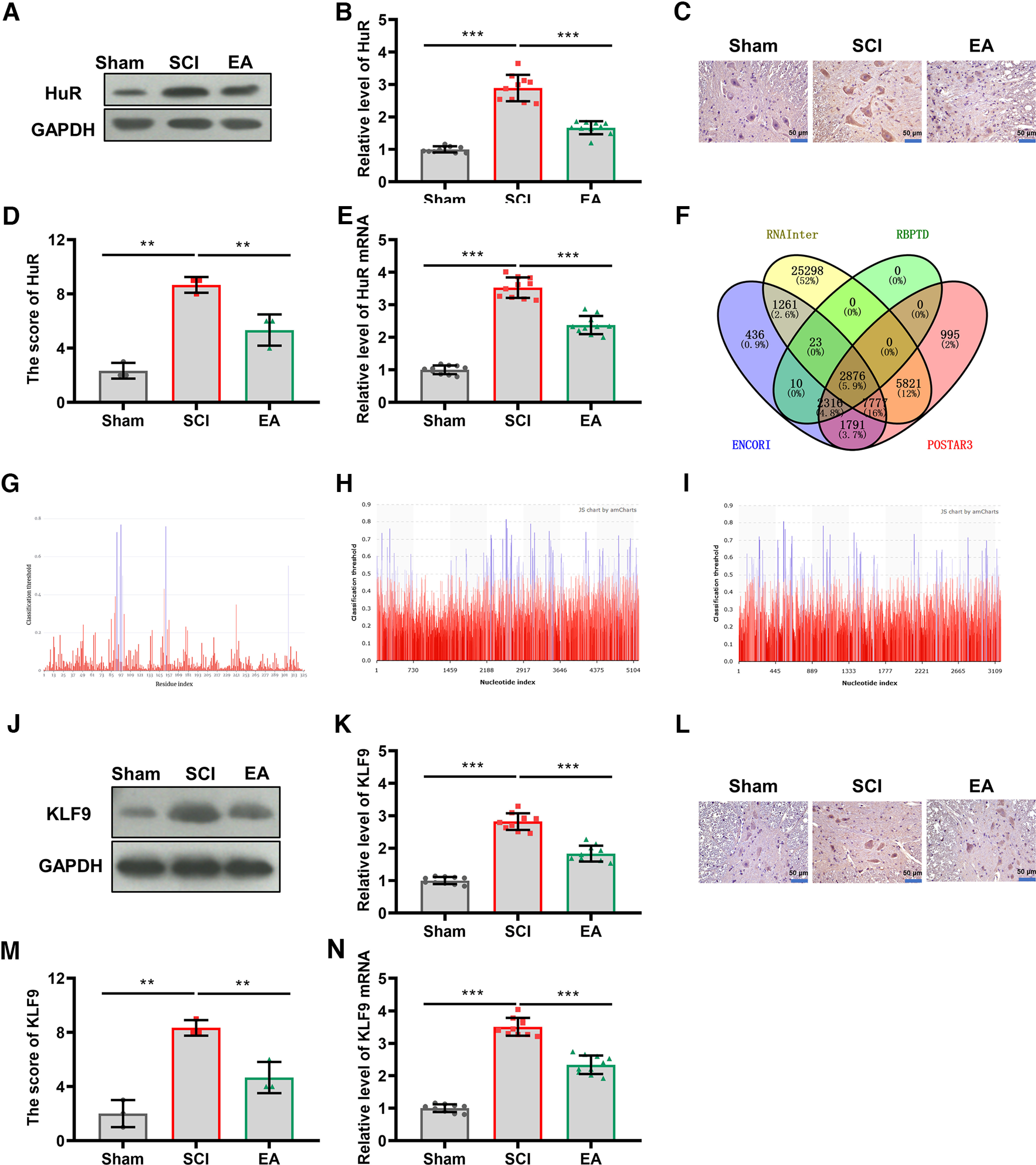
EA regulated HuR-mediated KLF9 expression. ***A***, HuR protein expression in the spinal cord tissues of the Sham, SCI, and EA groups (*N* = 10). ***B***, HuR protein levels in each group analyzed with one-way ANOVA followed by the Tukey’s test (*N* = 10). ***C***, The HuR protein expression was further verified with IHC in the spinal cord of injured sites (*N* = 3). ***D***, Statistical comparison of the scores of IHC (*N* = 3). ***E***, qRT-PCR was performed to measure HuR mRNA levels in the spinal cord tissues of SCI rats treated with or without EA (*N* = 10). ***F***, The intersection of mRNAs binding to HuR was predicted by four databases, including RNAInter, ENCORI, RBPTD, and POSTAR3. ***G–I***, The binding sites of HuR on KLF9 mRNA from *Homo sapiens* (***G***), *Mus musculus* (***H***), and *Rattus norvegicus* (***I***) were predicted using RNAInter. ***J***, Western blotting was performed to measure KLF9 expression in the spinal cord tissues of rats from each group (*N* = 10). ***K***, Statistical comparison of KLF9 protein was made in the rats after sham surgery, SCI operation, or EA treatment (*N* = 10). ***L***, The KLF9 protein expression was further verified with IHC in the spinal cord of injured sites, *N* = 3. ***M***, Statistical comparison of the scores of IHC (*N* = 3). ***N***, Relative expression of KLF9 mRNA was detected in spinal cord tissues of SCI rats treated with or without EA (*N* = 10). The *p* values were calculated with Tukey’s test for ***B***, ***D***, ***J***, and ***L***. ***p < 0.001.

Neuroinflammation after SCI has been the key etiology in secondary injuries (Gillespie and Ruitenberg, 2022). We explored the underlying mechanisms of EA-inhibited neurologic inflammation. Given the vital role of HuR in the progression and inflammation of SCI (Kwan et al., 2017b), the protein expression and mRNA level of HuR were evaluated via Western blot, immunohistochemistry (IHC), and qRT-PCR assays. Compared with the sham group, the SCI group showed a significant increase in HuR protein levels, whereas EA treatment reduced HuR protein expression (*p *<* *0.001, *n* = 10; Fig. 3*A*,*B*). The SCI-increased and EA-reduced HuR protein expression was further verified with IHC in the spinal cord of injured sites (Fig. 3*C*,*D*). A similar trend was observed in HuR mRNA by qRT-PCR analysis (*p *<* *0.001, *n* = 10; Fig. 3*E*). To determine the downstream target genes of HuR, four databases, including RNAInter, ENCORI, RBPTD, and POSTAR3, were used simultaneously to predict targets binding to HuR and found 2876 (5.9%) targets shared by all the databases (Fig. 3*F*). *KLF9* was speculated to be the target mRNA among all candidates, which promotes inflammation, oxidative stress, and apoptosis (Chen et al., 2022a). KLF9 has been reported to control the long-distance axon regeneration, and regulate the transcriptomes maintaining synaptic structural rearrangement and chronic pain following SCI (Apara et al., 2017; Mamet et al., 2017). Subsequently, PRIdictor (http://rnainter.org/PRIdictor/) from the RNAInter website was used for the prediction and showed that HuR could bind to the 3'UTR of KLF9 mRNA in human, mice, and rats (Fig. 3*G–I*). By examining KLF9 protein expression in spinal cord tissues, we discovered that the KLF9 protein level was significantly upregulated in the SCI group compared with the level in the sham group, and EA treatment significantly inhibited overexpression of KLF9 after SCI (*p *<* *0.001, *n* = 10; Fig. 3*J*,*K*). The SCI-increased and EA-reduced KLF9 protein expression was further verified with IHC in the spinal cord of injured sites (Fig. 3*L*,*M*). A similar inhibitory effect on overexpressed KLF9 mRNA was observed in qRT-PCR results (*p *<* *0.001, *n* = 10; Fig. 3*N*). Thus, we conjectured that EA may inhibit KLF9 mRNA stabilization by downregulating HuR expression post-SCI.

### HuR bound to KLF9 mRNA to promote the mRNA stability

**Figure 4.**
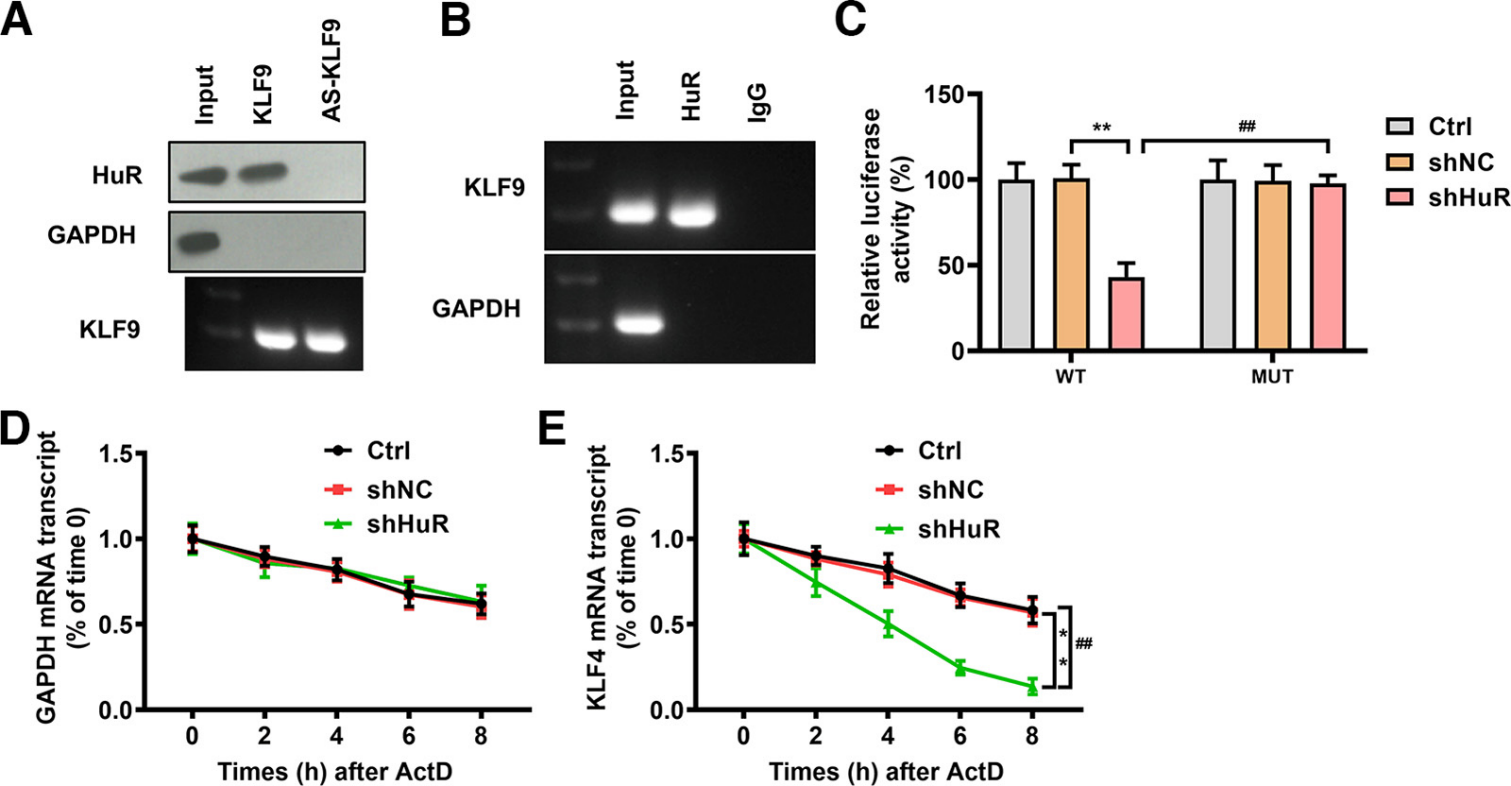
HuR bound to KLF9 mRNA to promote the mRNA stability. ***A***, RNA pull-down assay was performed to test the binding between HuR and KLF9 mRNA (*N* = 3). ***B***, The interaction between HuR and KLF9 mRNA was confirmed by RIP assay (*N* = 3). ***C***, 293T cells were cotransfected with luciferase reporter plasmids carrying the 3′ UTR of KLF9 mRNA, a control shRNA (shNC), or the shHuR. After 24 h, cells were lysed, and luciferase activities were measured. The *p* values were calculated with Tukey’s test (*N* = 3). ***D***, ***E***, The effect of HuR knockdown on the mRNA stability of GAPDH and KLF9 was determined by qRT-PCR after actinomycin D treatment (*N* = 3). The *p* values were calculated with Tukey’s test. **p < 0.01, ##*p* < 0.01.

Specific KLF mRNAs, such as KLF4, were predicted and verified as the direct targets of HuR (Zhou et al., 2009). To validate the binding of KLF9 mRNA to HuR, we performed an RNA pull-down assay in VSC4.1 cells. The assay results confirmed the direct interaction of HuR with KLF9 mRNA (Fig. 4*A*), which was supported by RIP assay using HuR antibody in VSC4.1 cells (Fig. 4*B*). To further determine the binding specificity, a luciferase reporter plasmid carrying the 3′ UTR of KLF9 mRNA with a putative HuR binding site was constructed. The results of the dual luciferase assay showed that transfection with shHuR significantly repressed the luciferase activity of the KLF9-WT vector (*p *<* *0.01, *n* = 3). No significant difference in luciferase activity was observed in the shHuR group compared with the shNC group, indicating the binding of HuR to the 3' UTR of KLF9 mRNA (Fig. 4*C*). To investigate the effect of HuR on KLF9 mRNA stability, we treated primary microglia with actinomycin D and found that shHuR could increase the half-life of KLF9 mRNA (*p *<* *0.01, *n* = 3; Fig. 4*D*,*E*). These findings demonstrated that HuR regulated KLF9 mRNA stability via direct binding to its 3′ UTR.

### HuR inhibition promoted EA-mediated neurologic repair after SCI

**Figure 5.**
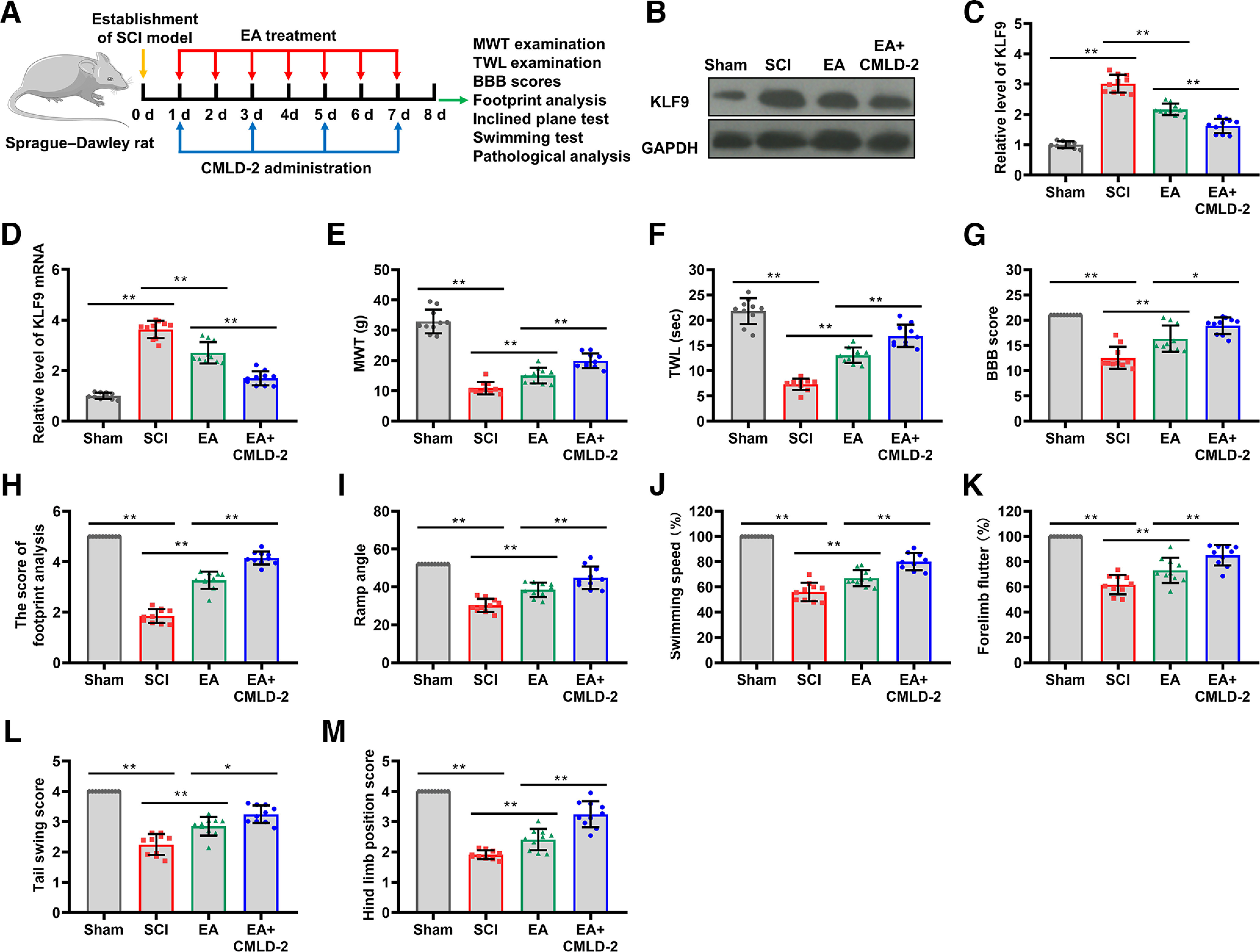
HuR inhibition promoted EA-mediated neurologic repair after SCI. ***A***, Flowchart of the EA or/and CMLD-2 treatments given to SCI rats. ***B***, After EA or/and CMLD-2 treatments given to SCI rats, the KLF9 protein expression in the spinal cord tissues was measured via Western blotting (*N* = 10). ***C***, Statistical comparison of KLF9 protein levels in each group (*N* = 10). ***D***, qRT-PCR was performed to measure KLF9 mRNA in spinal cord tissues of the rats treated with SCI, EA, or EA+CMLD-2 (*N* = 10). ***E***, ***F***, The effects of EA or/and CMLD-2 treatments on SCI-induced hyperalgesia were measured by MWT and TWL (*N* = 10). ***G***, BBB score was applied to assess the neurologic function of the SCI rats treated with EA or/and CMLD-2 (*N* = 10). ***H***, The movement stability and balance ability of the rats were assessed using the footprint test (*N* = 10). ***I***, The inclined plane test was performed, and the maximum angle was recorded in the Sham, SCI, and SCI groups with EA or/and CMLD-2 groups (*N* = 10). ***J–M***, The swimming performance of the rats in each group was evaluated by swimming rate (***J***), forelimb flutter (***K***), tail-swing score (***L***), and hindlimb position (***M***) to assess motor function recovery (*N* = 10). The *p* values were calculated with Tukey’s test for ***C–M***. *p < 0.05, and **p < 0.01.

Silencing HuR reduced the secretion of inflammatory cytokines, and inactivated inflammation signaling in microglia (Borgonetti and Galeotti, 2021). Similar effects could be achieved with SRI-42127 inhibition of HuR (Sorge et al., 2022). To verify EA-induced enhanced functional recovery after SCI via the HuR/KLF9 axis, treatment with a small-molecule inhibitor of HuR named CMLD-2 was given (Fig. 5*A*). KLF9 protein expression analyzed via Western blotting showed a significant increase in the SCI group compared with the sham group. EA stimulation decreased the overexpression of KLF9 protein, similar to the CMLD-2 treatment (*p *<* *0.01, *n* = 10; Fig. 5*B*,*C*). KLF9 mRNA expression was analyzed via qRT-PCR assay, and similar results were obtained (*p *<* *0.01, *n* = 10; Fig. 5*D*). We further researched the impact of HuR inhibition on pain thresholds in SCI rats. As shown in Figure 5, *E* and *F*, CMLD-2 efficiently facilitated increased TWL and MWT values mediated by EA (*p *<* *0.01, *n* = 10), suggesting that EA alleviated neuropathic pain through HuR inhibition. To investigate the effect of HuR inhibition on SCI repair, functional assessment based on BBB score, footprint test, and inclined plane test was evaluated in SCI rats. EA stimulation led to increased BBB score responses in SCI rats similar with CMLD-2 treatment, indicating that inhibition of HuR promoted recovery of motor function (*p *<* *0.01, *n* = 10; Fig. 5*G*). CMLD-2 was able to significantly enhance the effect of EA-induced neurologic repair, as evidenced by improvement in the footprint test scores (*p *<* *0.01, *n* = 10; Fig. 5*H*). The inclined plane test results also showed that CMLD-2 treatment enhanced EA-induced recovery of motor function in SCI rats (*p *<* *0.01, *n* = 10; Fig. 5*I*). A swimming test was conducted to evaluate the effect of HuR inhibition on motor function recovery post-SCI. The results demonstrated that rats in the EA+CMLD-2 group had a significantly higher (10%) swimming rate than EA-treated rats (*p *<* *0.01, *n* = 10; Fig. 5*J*). CMLD-2 administration was also beneficial in improving EA-induced higher forelimb flutter frequency (*p *<* *0.01, *n* = 10; Fig. 5*K*). Simultaneous treatment with CMLD-2 and EA markedly increased tail swing scores as well as hindlimb position scores in SCI rats compared with only EA treatment (*p *<* *0.01, *n* = 10; Fig. 5*L*,*M*). Overall, the inhibition of HuR with CMLD-2 could enhance EA-mediated neurologic repair after SCI.

### HuR inhibition relieved EA-mediated inflammation after SCI

**Figure 6.**
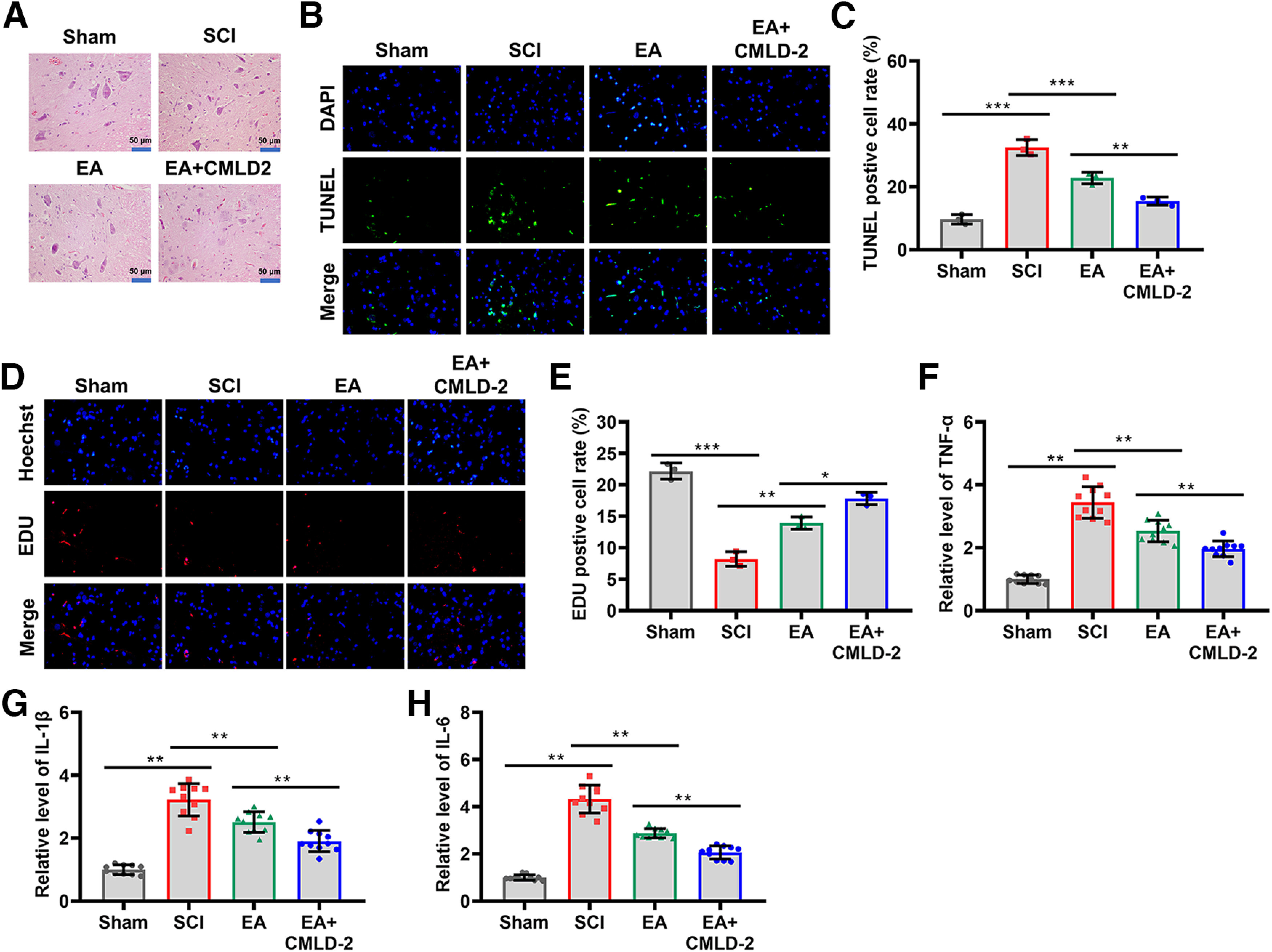
CMLD-2 enhanced EA-mediated inflammation inhibition of SCI rats. ***A***, H&E staining was conducted on spinal cord sections of the Sham, SCI, EA, and EA+CMLD-2 groups. Scale bar, 50 µm (*N* = 3). ***B***, TUNEL assay was performed to evaluate the effects of EA combined with CMLD-2 on cell apoptosis. Scale bar, 40 µm (*N* = 3). ***C***, Statistical comparison of the levels of TUNEL-positive cells (*N* = 3). ***D***, EDU staining was used to detect cell proliferation in spinal tissue sections from each group (*N* = 3, ×100). ***E***, Statistical comparison of the levels of EDU-positive cells (*N* = 3). ***F–H***, The effects of EA+CMLD-2 treatments on the levels of TNF-α (***F***), IL-1β (***G***), and IL-6 (***H***) in spinal cord tissues were assessed via ELISA (*N* = 10). The *p* values were calculated with Tukey’s test for ***C–E***. **p < 0.01.

H&E staining was used to examine the pathologic effects of EA+CMLD-2 treatment on spinal cord tissues. We observed a significant reduction in damage in numerous tissue sections (Fig. 6*A*). As demonstrated by TUNEL staining, CMLD-2 treatment was partially responsible for the beneficial effect of EA on neurons apoptosis (Fig. 6*B*,*C*). Furthermore, EDU staining showed that CMLD-2 administration was beneficial in enhancing EA-induced neuronal proliferation (Fig. 6*D*,*E*). The results of ELISA revealed that CMLD-2 administration significantly enhanced the anti-inflammatory effect of EA. Additionally, it showed a synergistic effect on the downregulation of SCI-induced increased TNF-α, IL-6, and IL-1β levels (*p *<* *0.01, *n* = 10; Fig. 6*F–H*). These findings demonstrated that HuR inhibition relieved EA-mediated inflammation after SCI.

## Discussion

In the present study, we discovered that EA treatment improved the recovery of motor function and pain thresholds by relieving inflammation in rats with SCI. Based on this finding, we further demonstrated that EA administration inhibited KLF9 mRNA stability by downregulating HuR expression. Interestingly, inhibition of HuR-ARE interaction enhanced EA-mediated neurologic repair in SCI rats. The investigation into the underlying mechanisms expounded that EA treatment enhanced neurologic repair, which expands the knowledge of EA in SCI treatment.

EA therapy is widely known for its effectiveness in improving motor and sensory functions in rats with SCI, which is beneficial for neurologic recovery (Ding et al., 2011; Tan et al., 2022). Our results showed that EA treatment notably increased pain thresholds and improved the movement stability of SCI rats, confirming the positive impact of EA treatment on SCI. Inflammation plays a prominent role in neurodegenerative conditions like SCI, where a rapid and robust upregulation of proinflammatory cytokines, including TNF-α, IL-6, and IL-1β, is reported to trigger apoptotic cell death (Kim et al., 2017). Furthermore, Xu et al. (2020) determined the role of TLR4 in relieving inflammation in spinal cord injury by detecting changes in TNF-α, IL-6, and IL-1β levels. Our results showed that EA treatment inhibited neuronal apoptosis and partially rescued SCI-mediated upregulation of inflammatory factors and Caspase-3 activation, indicating the neuroprotective effects of EA mediated by anti-inflammation. These findings were consistent with previous reports showing the protective effects of MA at Shuigou (GV26) and Yanglingquan (GB34; Choi et al., 2010). Instead of MA, EA treatment was used in our study, which has been reported to be more effective (Jiang et al., 2014). Unlike previous studies, two acupoints, including Baihui (GV20) and Jiaji (Ex-B2), were also added in the present study, expanding the role of EA and providing a new treatment strategy for SCI.

Although the role of EA in neurologic recovery after SCI is known, its underlying mechanism remains unknown. Growing evidence has pointed to the notion that HuR, an RNA binding protein, plays a pivotal role in nerve regeneration, apoptosis, and various diseases related to the CNS (Sorge et al., 2022; Zhou et al., 2022a). The study Kwan et al. (2017b) shows that HuR is translocated from the nucleus to the cytoplasm in the acute phase of SCI, further accentuating neuronal injury. HuR expression is activated in spinal cord injury and positively regulates the molecular response of key inflammatory mediators in astrocytes (Kwan et al., 2017a). Additionally, Wang et al. (2023) found that silencing HuR increased the Basso, Beattie, and Bresnahan score, relieved SCI, and decreased neuronal-like apoptosis. Therefore, we believe that HuR participated in the pathologic process of EA to reduce SCI. However, the correlation between EA and HuR expression in SCI remains largely unknown. Hence, we explored the contribution of HuR in the EA-induced neurologic repair. We found that HuR was overexpressed in spinal cord tissues of SCI rats and that EA treatment attenuated this increase, implying that HuR expression was tightly regulated by EA in neurologic repair. Electrical stimulation is the major basis of EA application and could form an electric field of the appropriate intensity and time (Ridding and Rothwell, 2007). The electric field response induces bidirectional axon growth across the damaged cord region via cathodal attraction in SCI therapy (Li, 2019). SOX2 is a member of the SOX gene family and is involved in NSC differentiation. Direct cortical stimulation by a pulsed electric field with 100 Hz induces SOX2 expression in cortical neural precursor cells (Chang et al., 2016), and HuR has been reported to bind SOX2 mRNA (Galardi et al., 2020). Therefore, the mechanism of EA-mediated HuR regulation might involve the SOX2 pathway, which might be studied further by us. In a nutshell, our research provided evidence for the involvement of a HuR-related mechanism in SCI treatment by EA.

Next, we determined the potential downstream targets of HuR predicted via databases and found a lot of candidates, including KLF9. In recent years, the regulatory role of KLF9 in inflammation and neuronal apoptosis has received enthusiastic attention. KLF9 is a member of the KLF family and plays an important role in neurologic disorders. The research results of Ephraim F. Trakhtenberg show that KLF9 suppression boosts axon growth after optic nerve injury (Apara et al., 2017). Independent work in the spared nerve injury model of chronic pain has shown that decoy AYX2 binds to KLF9 mRNA and causes a significant reduction in mechanical hypersensitivity (Mamet et al., 2017). Notably, KLF9 was found to be involved in inflammatory response. Results from the study by Qu et al. (2022) reveal that downregulation of KLF9 ameliorates LPS-caused acute lung injury and inflammation in mice. These findings underscore that KLF9 may be a potential target in nerve injury. Hence, we analyzed KLF9 protein and KLF9 mRNA levels in SCI rats via Western blotting and qRT-PCR. We found higher levels of KLF9 protein and mRNA similar with the prior studies demonstrating that KLF9 is overexpressed in the spinal cord under pathologic nociceptive conditions (Nilsson et al., 2005; Fu et al., 2009). But we went one step further with the identification of HuR binding to KLF9 mRNA in SCI by *in vitro* experiments. The results obtained from these studies supported the hypothesis that EA inhibits KLF9 mRNA stabilization by downregulating HuR expression as a regulatory mechanism of EA treatment in SCI. In addition, *KLF9* might be used as a promising molecular target for SCI therapy clinically.

HuR has the ability to modulate inflammatory responses in renal injury (Chen et al., 2022b), muscular dystrophy (Mubaid et al., 2019), and intervertebral disk degeneration (Shao et al., 2020). The increased secretion of inflammatory cytokines in the acute phase of SCI plays an important role in tissue damage. HuR is activated during the early phase of CNS trauma and regulates the molecular response of key inflammatory mediators in astrocytes (Kwan et al., 2017a). In the current study, we found that inhibition of HuR by EA had a synergistic reversing effect on SCI-induced upregulation of TNF-α, IL-6, and IL-1β cytokines, indicating alleviation of inflammation by EA-mediated HuR inhibition. Additional evidence has emerged to suggest that KLF9 could regulate inflammation. An enhanced proinflammatory state has been observed in the liver of Klf9^−/−^mice fed a high-fat diet (Brown et al., 2022). Reduced levels of TNF-α, IL-1β, and IL-6 during KLF9 silencing suggest suppressed inflammation in gestational diabetes mellitus (Chen et al., 2022a). A binding relationship between the HuR and KLF9 was identified in this study, suggesting that HuR/KLF9 axis is involved in EA-mediated inflammation in SCI. These findings provide potential therapeutic targets for the treatment of SCI-related inflammation.

Finally, a series of i*n vivo* experiments were conducted, and HuR inhibitor CMLD-2 was used to study the regulatory relations among EA, HuR, and KLF9 mRNA in SCI. EA stimulation strongly repressed SCI-induced KLF9 overexpression, and HuR inhibitor CMLD-2 enhanced this EA-mediated inhibiting effect. Our data uncovered, for the first time, the contribution of the HuR/KLF9 axis in the progression of SCI. In addition, *in vivo* CMLD-2 administration was found beneficial in improving EA-mediated increased TWL and MWT values, BBB scores, footprint test scores, swimming rates, and forelimb flutter frequencies. These results highlight EA-enhanced neurologic repair after SCI via the HuR/KLF9 axis, enhance our understanding of EA-mediated recovery post-SCI, and open novel avenues for potential therapeutic intervention in SCI. In our study, we have also confirmed the regulation of KLF9 mRNA by HuR. Interestingly, it has been proven that KLF8 (another transcription factor of the KLF family) regulates the expression of HuR (Govindaraju and Lee, 2014). Similarly, there may be a regulatory correlation between HuR and KLF9 in SCI, which could provide new insights for the treatment of SCI.

Notably, we acknowledge the following limitations of the study: (1) the study was specific to binding mRNAs, and only KLF9 was verified, and, thus, validation of other mRNAs is needed; (2) in the experiment exploring the neural function recovery of the SCI animal model, a single time point on day 7 post-SCI was studied, and additional time points are warranted to detect time-dependent variations; and (3) more potential upstream regulators of HuR expression need to be discovered.

Overall, the data obtained in this study showed that EA enhances nerve repair and reduces inflammation after SCI, and provided a novel regulatory axis: HuR/KLF9. Our study provides insights into new molecules for nerve repair after SCI.

## Synthesis

Reviewing Editor: Eran Perlson, Tel Aviv University Decisions are customarily a result of the Reviewing Editor and the peer reviewers coming together and discussing their recommendations until a consensus is reached. When revisions are invited, a fact-based synthesis statement explaining their decision and outlining what is needed to prepare a revision will be listed below. The following reviewer(s) agreed to reveal their identity: NONE.

The authors answer most of the reviewers concerns but in order to be published in eNeuro they must provide also the data demonstrating EA effect on neuroregeneration and not keep it for future publication. it is essential to show that EA improves motor function due to neuroregeneration.

## Author Response

In this study, the authors investigated the underlying mechanism of EA in neurological repair after SCI. First, they investigated the contribution of EA in the neurological repair of the SCI rat model. The expression levels of Hu antigen R (HuR) and Kruppel-like factor 9 (KLF9) in spinal cord tissues were quantified after EA treatment. Second, they carried out bioinformatics analysis, RNA pull-down assay, RNA immunoprecipitation, and luciferase reporter gene assay to verify the binding of HuR and KLF9 mRNA for mRNA stability. Last, HuR inhibitor CMLD-2 was used to verify the enhanced effect of EA on neurological repair after SCI via the HuR/KLF9 axis.

(1) The claim that EA improved motor function is only supported by behavioral outcomes. Improved motor function is primarily due to neuroregeneration and or neurogenesis, which has not been explored in this study.

Response: Thank you very much for the suggestions provided by the reviewer, which are of great significance in improving the quality of the manuscript. The comments made by the reviewer align with our thoughts. Recently, we have been exploring the effects of electroacupuncture combined with small molecule drug (a type of traditional Chinese medicine extract) on nerve regeneration, and have found that extracellular vesicles play an important role in this process. Considering that the research results of the effect of electroacupuncture on nerve regeneration will affect the publication of our follow-up research Therefore, our research findings will be published in the future.

(2) Also, the emphasis on HuR and KLF9 needs to be justified and their expression in different cell types is not clear. More immunohistochemical data about the cellular expression of HuR and KLF9 may support the conclusion.

Response: Thank you very much to the reviewer for their suggestions. We further emphasize the importance of HuR and KLF9 in this study. We have already conducted IHC detection of HuR and KLF9 expression in rat spinal cord tissue before and after EA treatment. Additionally, WB detection has also been performed. Our data sufficiently demonstrates that EA can influence the expression of HuR and KLF9. However, due to the inability to directly manipulate EA on cells, it was not possible to detect changes in HuR and KLF9 expression at the cellular level through IHC detection.

*Major concerns*

(3) The manuscript is full of English and grammar errors. Rigorous revision is needed for the entire text.

Response: We greatly appreciate your effort and helpful comments for improving our manuscript. We have carefully checked and tried our best to present a more accurate and excellent paper.

(4) Methods:

• The source of motoneurons cells is not provided (not clear if this is a vendor or a gift from collaborator).

Response: We apologized that we did not clearly clarify this part. We have added cell sources and product numbers in the Methods section.

• Missing sections for perfusion, tissue processing, and sectioning must be provided.

Response: Sections for perfusion, tissue processing, and sectioning have provided in supplemental.

• In line 77, the statement “All cells were incubated at 37”, do you have another cell line in the study?

Response: We apologized that we did not clearly clarify this part. We have redescribed this section.

• All sections in the method should start with the goal or aim of the used technique.

Response: We greatly appreciate your effort and helpful comments for improving our manuscript. We added the goal or aim of the used technique in the methods section.

• For the “Analysis of mRNA binding to HuR” section, please provide more details about goals of the experiment and the procedure.

Response: For the “Analysis of mRNA binding to HuR” section, we predicted the downstream targets of HuR by bioinformatics analysis rather than experimentation. We have also added more information on bioinformatics analysis.

• In the dual-luciferase assay, what type of cells were transfected?

Response: We performed dual-luciferase assay with 293T cells. We checked the full manuscript and corrected the incorrect description.

(5) Results:

• Figure 2A, H and E showed no difference between the three images.

Response: Thank you for your attention and efforts. We checked the images and found that we uploaded the wrong ones. We have updated the correct images.

• Statistical analysis is not described in figure legends for each specific graph of all quantitative measurements. Also, analysis was not appropriate (t-test vs ANOVA).

Response: Thank you for your efforts and pointing out the shortcomings of our manuscript. Statistical analysis is added in figure legends for each specific graph of all quantitative measurements. We checked the statistical analysis and corrected incorrect statistics.

• Figure 2, Scale bar is not present in TUNEL stain. Also, quantitative measurement of TUNEL-positive cells is important to validate the observation.

Response: Thank you for pointing that out. We apologize that we do not clearly clarify this part. We supplemented the magnification of TUNEL staining. In addition, we added quantitative analysis results of TUNEL staining.

• Figure 2D, it is unclear how the Caspase-3 graph was established; all these points are from Western? The n number of experimental groups to run statistical analysis must be provided in D. Also, the original blots of all experiments should be provided at least in supplemental.

Response: We examined the gray values of the proteins in Figure 2C and thus calculated the relative levels of the proteins. Additionally, we added the n number of experimental groups. The original blots of all experiments have provided in supplemental.

• Figure3 A & B, and I& J, the same issue as above. N of experiments should be provided in the figure legend and original blots must be provided in supplemental.

Response: Thank you to the reviewers for their meaningful comments. The N of experiments has provided in the figure legend and original blots have provided in supplemental.

• Figure 3C&K, quantitative statistical analysis of IHC must be provided.

Response: Thank you to the reviewers for their meaningful comments. The quantitative statistical analysis of IHC have provided.

• Figure 5B, the N of experiments for quantitative analysis of Western blot proteins should be provided. Also, original blots should be provided in supplemental.

Response: Thank you to the reviewers for their meaningful comments. The N of experiments has provided in the figure legend and original blots have provided in supplemental.

• Figure 5C, t-student test is not suitable. Also, clarify the method used for statistical analysis in all graphs.

Response: Thank you for your efforts and pointing out the shortcomings of our manuscript. We modified inappropriate statistical methods. Student’s t-test was used to analyze the differences between two groups. One-way ANOVA analysis followed by the Tukey test was used for multi-sample analysis.

• Figure 6, please clarify the method used for statistical analysis in all graphs.

Response: Thank you for your helpful comments. We clarified the method used for statistical analysis in all graphs.
